# Prevalence and experiences of presenteeism in the workplace: a cross-sectional survey of an English National Health Service Mental Health Trust

**DOI:** 10.3389/frhs.2026.1823661

**Published:** 2026-07-08

**Authors:** Jennifer Sweetman, Mona Kanaan, Sarah Morris, Helen Cooke, Angela Collins, Emily Shoesmith, Elena Ratschen

**Affiliations:** 1 Department of Health Sciences, University of York, York, United Kingdom; 2 Tees, Esk and Wear Valleys NHS Foundation Trust, Middlesbrough, United Kingdom

**Keywords:** long-term conditions, NHS, presenteeism, survey, wellbeing, workforce

## Abstract

**Introduction:**

Presenteeism (working while unwell) is prevalent in the English National Health Service (NHS) workforce and is associated with substantial costs. Currently little is known about the relationship between presenteeism and staff wellbeing within the NHS. To identify the proportion of employees working while unwell and how often this occurs, assess differences in views about presenteeism where long-term conditions and line management responsibilities play a role, and to explore how affected individuals might be identified and supported within the NHS workforce.

**Method:**

An online survey with employees of a large NHS Mental Health Trust (9,852 employees) based in the North of England (February-March 2025). Questions were multiple choice or free text. Numerical data were analysed descriptively; text data were analysed thematically.

**Results:**

In total, 1,828 (18.6%) employees consented to complete the survey; 80%(*n* = 1,462) were female and the largest group (*n* = 545; 29.8%) were aged 51–65 years. Most(88.9%, *n* = 1,579) indicated presenteeism at least twice in the three-months before completing the survey, 68.3%(*n* = 1,168) considered that presenteeism was relevant or very relevant to their team, and 53.9%(*n* = 922) indicated that presenteeism is expected or common and accepted in their team. Statistically significant differences in survey responses were found between individuals with and without long-term conditions.

**Discussion:**

Employee wellbeing and presenteeism research is relevant, acceptable and of interest to NHS employees. Further research focusing on supporting employees working with long-term conditions is warranted.

## Introduction

The National Health Service (NHS) workforce is crucial to the delivery of healthcare to the UK population. However, despite recent increases to staffing levels (1) and investment to tackle workforce challenges (2), NHS targets are largely not being met (3, 4). This may be the result of a healthcare system which is under strain, with consequences for workforce wellbeing (5). The Nuffield Trust highlighted that sickness absence levels within the NHS were rising substantially (4.3% in 2019, 5.6% in 2022) and estimated that 27 million days were lost to sickness absence (equating to 74,500 full-time staff members) in 2022 (6). In addition to absence from the workplace, the British Medical Association and the wider research literature have indicated that a culture of presenteeism (attending work while unwell) is also affecting the NHS (7–9).

In the 2024 NHS staff survey, data collected from over 700,000 NHS employees across England indicated that over half of respondents had attended work while unwell in the previous three months; this rate of presenteeism has not reduced since 2020 (10). The impact of presenteeism on organisations is challenging to calculate; however, the literature suggests there may be a greater cost associated with presenteeism than with absenteeism (11–14). The 2025 report from the Health Foundation aligns with this, highlighting the decline in health among working age people and the impact of ill-health among the UK workforce [including lost productivity (presenteeism), sickness absence and recruitment costs]. Report authors indicated a cost of up to £150bn to UK employers from ill health in the workforce (15).

Presenteeism has been associated with mental and physical health issues (9, 14, 16–18). Within England, research suggests that there is a North-South divide, with higher rates of mental and physical health inequalities in the North of England (19, 20). This research explores the experiences of presenteeism and wellbeing in staff employed by a large NHS mental Health Trust (9,852 employees) based in the North of England. NHS Staff survey data indicates a high rate of presenteeism (53.5% attended work when unwell in the 3 months leading to NHS Staff Survey completion, national average 55.7%) within the Trust workforce, coupled with a higher rate of long term mental or physical health conditions than the national average (35.1% reported having a physical or mental health problem lasting/expected to last for 12 months or more, national average 25.2% (10). This Trust is committed to promoting health and wellbeing within their workforce and have received recognition for efforts towards improving health at work (21); this research presented an opportunity to explore the impact of presenteeism from the perspective of employees and the organisational support structures which could influence employee decisions about attending work while unwell.

Within the context of a single NHS Mental Health Trust, we aimed to conduct a survey of employees to:

- Identify the proportion of employees who attend work while unwell and how often this occurs,
- Assess whether there are differences in views about, and the frequency of self-reported presenteeism as indicated by (a) people with and without a long-term condition and (b) those with and without line management responsibilities,
- Explore how presenteeism might be identified within NHS workforce settings, and how employees can be supported.

## Materials and methods

### Study design

A cross-sectional mixed-methods survey was conducted between February and March 2025. Details of this study are reported following the STROBE guidelines for cross-sectional studies [the STROBE checklist can be found in the Supplementary Material: S1 (22)].

### Setting, participants and sample size

All employees of an NHS Mental Health Trust based in the North of England were invited to participate in the survey; given their employed status capacity to consent to participate in this survey was assumed. At the time of data collection, the Trust employed 9,852 people working in a variety of roles including but not limited to: clinical, scientific/technical, management, administration and central functions. No other inclusion or exclusion criteria were applied.

### Recruitment and bias

A variety of methods to raise awareness of the survey were used to reduce bias: member of the NHS Trust Research and Development team sent a standardised email to all employees of the Trust inviting them to participate in the survey; a link to the survey participant information and main survey contents was included in the email. Additionally, the following Trust communication methods were used to promote the survey: staff newsletters, social media accounts and wellbeing champions promoted the survey, and posters including a QR code were placed in communal areas of the Trust to inform employees working in roles which did not involve computers about the survey. After following the survey link, individuals were required to provide informed consent to participate before the survey content was displayed. Those who completed the survey were given the option to leave contact details if they wished to be entered into a prize draw or be contacted about further research about presenteeism and staff wellbeing.

### Data: variables, sources and measurement

A survey, hosted on the online Qualtrics survey platform, was developed by the research team in collaboration with the study NHS partners. After responding to the mandatory consent question, all survey questions were optional with reminder prompts associated with demographic questions to minimise missing data related to the sample characteristics. No validated questionnaires were included in the survey content.

This survey was organised into four sections comprising demographic information, experiences of presenteeism, noticing presenteeism in the workplace, and support options relating to presenteeism in the workplace. In total, 28 main questions were presented to respondents, with additional sub-questions presented to collect further details relating to seven of these main questions; this survey represents a convergent parallel mixed-methods design (23). Questions either asked respondents to select a response from a list of available options (quantitative, categorical data) or to provide a free-text response (qualitative data). Full details of the survey questions and response options for each question are shown in the Supplementary Material (S2). Variables identifying the presence or absence of a long-term condition (defined as a condition that cannot at present be cured but can be controlled by medication and therapies) and of line-management responsibilities were used to understand survey responses, in addition to the analysis of the full sample responses.

### Data analysis: statistical and qualitative methods

Descriptive statistics were provided. Categorical variables were summarised by frequency (*n*) and percentage (%) of total responses per category. These statistics were analysed for the full sample, and by long-term condition and line-management responsibilities.

Unadjusted differences in categorical variables' responses by those with and without a long-term condition were assessed using Chi-squared or Fisher's Exact statistics. A similar approach was used for assessing association of these variables with line-management responsibilities. For these analyses, the response options “prefer not to say” and “other” for these questions were not included in the analysis. Missing values were also excluded from these analyses.

Post-hoc subgroup analyses were conducted following analysis of qualitative free-text responses which suggested that there might be differences in views relating to experiences of presenteeism depending on age. Due to small numbers in the lowest (16–20 and 21–30) and highest age categories (51–65 and 65+), age was recategorised into four categories (≤30 years, 31–40, 41–50, >50).

Given the exploratory nature of this study, a significance level of *p* < 0.01 was applied for all subgroup analyses. Confidence intervals are also provided for these estimates. SPSS Version 29.0.2.0 was used to complete the statistical analysis.

Qualitative responses were imported into Microsoft Excel, organised thematically and summarised incorporating responses from the full sample. In total, 13 questions in the survey enabled free-text responses. A pragmatic approach was used to systematically organise the data, initially based on the survey questions (deductive coding) and then by the main topic included in the response (inductive coding); for example, when considering the survey question asking respondents to share how their work had been affected by presenteeism responses covered many topics including: work adaptations, team dynamics, incomplete tasks, comparisons between people with and without long-term illnesses, concerns about attendance records, concerns about becoming ill, safe staffing levels. Responses included under similar codes were combined across the dataset to generate themes. Summaries of the responses captured by each theme (including relevant responses as quotes) are presented in the context of the quantitative analysis to better understand the quantitative findings (narrative integration; 23). All analysis work was completed by one researcher, supported by a second researcher. Differences in opinion about the presentation of results, or the organisation of qualitative responses were resolved through discussion.

### Ethics

This study received approval from the NHS Health Research Authority and Health and Care Research Wales in January 2025 (REC reference: 24/HRA/5300). Capacity to consent was assumed for all organisational employees; the survey was voluntary and non-invasive. All participants confirmed that they had read study information and consented to participate; all responses were anonymous.

## Results

### Sample characteristics

In total, 1,828 (18.6%) NHS Trust employees consented to participate in the survey. After providing consent, all responses were voluntary and therefore the response rate varied per question. For the primary questions (shown to all survey respondents), responses were received from between 1,447 and 1,818 participants, with the exception of questions relating to current presenteeism support which were completed by between 562 and 1,014 respondents. This difference in response rate for these questions could relate to the specific wording of these questions, or the framing of current support offered by the NHS Trust which may not be clearly linked to presenteeism. All 1,828 participants provided at least one free-text response to the qualitative questions.

Tables 1, 2 present the demographic and occupational characteristics of the sample. The diversity of respondents within this sample demonstrates the motivation of employees across all service types and occupational groups within the Trust to participate in research of this nature.

**Table 1:** Demographic characteristics (*N* and percentage) of total sample and by presence of long-term conditions and whether in a line management role.

Characteristic	Whole sample	Role involves LM	Role does not involve LM	*p* values	Reports long-term condition	No long-term condition	*p* values
Number of respondents *N*	1,828	462	1,310		753	944	
Gender *N* (%)							
Female	1,462 (80.0)	364 (78.8)	1,069 (81.6)	<0.001	597 (79.3)	768 (81.4)	0.616
Male	337 (18.4)	90 (19.5)	232 (17.7)		148 (19.7)	169 (17.9)	
Non-binary	3 (0.2)	2 (0.4)	1 (0.1)		2 (0.3)	1 (0.1)	
Self-describe	1 (0.1)	0 (0)	1 (0.1)		0 (0)	1 (0.1)	
Prefer not to say	15 (0.8)	6 (1.3)	7 (0.5)		6 (0.8)	5 (0.5)	
Missing	10 (0.5)	0 (0)	0 (0)		0 (0)	0 (0)	
Age *N* (%)							
16–20	9 (0.5)	1 (0.2)	7 (0.5)	<0.001	0 (0)	8 (0.8)	<0.001
21–30	298 (16.3)	33 (7.1)	256 (19.5)		95 (12.6)	179 (19.0)	
31–40	464 (25.4)	111 (24.0)	343 (26.2)		163 (21.6)	272 (28.8)	
41–50	458 (25.1)	157 (34.0)	292 (22.3)		206 (27.4)	227 (24.0)	
51–65	545 (29.8)	152 (32.9)	380 (29.0)		278 (36.9)	233 (24.7)	
66+	28 (1.5)	1 (0.2)	26 (2.0)		9 (1.2)	17 (1.8)	
Prefer not to say	15 (0.8)	7 (1.5)	6 (0.5)		2 (0.3)	8 (0.8)	
Missing	11 (0.6)	0 (0)	0 (0)		0 (0)	0 (0)	
Ethnicity *N* (%)							
White	1,668 (91.2)	430 (93.1)	1,202 (91.8)	0.002	716 (95.1)	847 (89.7)	<0.001
Mixed or multiple ethnic background	23 (1.3)	6 (1.3)	17 (1.3)		11 (1.5)	11 (1.2)	
Asian or Asian British	36 (2.0)	4 (0.9)	29 (2.2)		11 (1.5)	21 (2.2)	
Black or African or Caribbean or Black British	66 (3.6)	17 (3.7)	46 (3.5)		10 (1.3)	52 (5.5)	
Other	6 (0.3)	0 (0)	6 (0.5)		1 (0.1)	4 (0.4)	
Prefer not to say	18 (1.0)	5 (1.1)	10 (0.8)		4 (0.5)	9 (1.0)	
Missing	11 (0.6)	0 (0)	0 (0)		0 (0)	0 (0)	
Long-term condition							
Yes	753 (41.2)	185 (40.0)	558 (42.6)	0.003	753 (100)	0 (0)	–
No	944 (51.6)	253 (54.8)	682 (52.1)		0 (0)	944 (100)	
Prefer not to say	30 (1.6)	7 (1.5)	19 (1.5)		0 (0)	0 (0)	
Missing	101 (5.5)	17 (3.7)	51 (3.9)		0 (0)	0 (0)	

LM, line management.

**Table 2:** Occupational characteristics (*N* and percentage) of total sample and by presence of long-term conditions and whether in a line management role.

Characteristic	Whole sample	Role involves LM	Role does not involve LM	*p* values	Reports long-term condition	No long-term condition	*p* values
Number of respondents *N*	1,828	462	1,310		753	944	
Service *N* (%)							
Inpatient	443 (24.2)	130 (28.1)	302 (23.1)	0.005^a^	179 (23.8)	235 (24.9)	0.188^a^
Outpatient	124 (6.8)	29 (6.3)	88 (6.7)		50 (6.6)	67 (7.1)	
Community	990 (54.2)	228 (49.4)	747 (57.0)		401 (53.3)	528 (55.9)	
Office-based	453 (24.8)	137 (29.7)	306 (23.4)		207 (27.5)	228 (24.2)	
General buildings and estates	38 (2.1)	10 (2.2)	28 (2.1)		19 (2.5)	15 (1.6)	
Prefer not to say	35 (1.9)	11 (2.4)	16 (1.2)		14 (1.9)	10 (1.1)	
Missing	50 (2.7)	11 (2.4)	17 (1.3)		14 (1.9)	10 (1.1)	
Occupational group							
Allied Health Professionals^b^	399 (21.8)	82 (17.7)	315 (24.0)	<0.001^a^	138 (18.3)	237 (25.1)	0.190^a^
Medical and dental	71 (3.9)	25 (5.4)	46 (3.5)		28 (3.7)	38 (4.0)	
Ambulance (operational)	1 (0.1)	0 (0)	1 (0.1)		0 (0)	1 (0.1)	
Public health	28 (1.5)	4 (0.9)	24 (1.8)		13 (1.7)	14 (1.5)	
Commissioning	1 (0.1)	0 (0)	1 (0.1)		0 (0)	1 (0.1)	
Registered nurses and midwives	423 (23.1)	156 (33.8)	258 (19.7)		182 (24.2)	218 (23.1)	
Nursing or healthcare assistants	370 (20.2)	68 (14.7)	296 (22.6)		156 (20.7)	192 (20.3)	
Social care	47 (2.6)	8 (1.7)	38 (2.9)		21 (2.8)	21 (2.2)	
Wider Healthcare Team^c^	466 (25.5)	125 (27.1)	333 (25.4)		215 (28.6)	231 (24.5)	
Prefer not to say	51 (2.8)	9 (1.9)	33 (2.5)		18 (2.4)	23 (2.4)	
Missing	77 (4.2)	10 (2.2)	37 (2.8)		19 (2.5)	26 (2.8)	
Length of service							
Less than 1 year	173 (9.5)	21 (4.5)	148 (11.3)	<0.001	55 (7.3)	109 (11.5)	0.002
1–5 years	688 (37.6)	125 (27.1)	552 (42.1)		284 (37.7)	364 (38.6)	
6–9 years	275 (15.0)	77 (16.7)	198 (15.1)		116 (15.4)	148 (15.7)	
10 + years	650 (35.6)	235 (50.9)	407 (31.1)		298 (39.6)	317 (33.6)	
Prefer not to say	8 (0.4)	2 (0.4)	4 (0.3)		0 (0)	6 (0.6)	
Missing	34 (1.9)	2 (0.4)	1 (0.1)		0 (0)	0 (0)	
Line Management							
Yes	462 (25.3)	462 (100)	0 (0)	–	185 (24.6)	253 (26.8)	0.460
No	1,310 (71.7)	0 (0)	1,310 (100)		558 (74.1)	682 (72.2)	
Prefer not to say	25 (1.4)	0 (0)	0 (0)		10 (1.3)	9 (1.0)	
Missing	31 (1.7)	0 (0)	0 (0)		0 (0)	0 (0)	

LM, line management.

a Respondents who selected multiple options for this variable were excluded from the significance calculation as there was no indication of a primary response category (Service *n* = 202, Occupational group *n* = 45);

b Allied Health Professionals or Healthcare Scientists or Scientific and Technical;

c Wider Healthcare Team includes admin and clerical, central functions and corporate services, maintenance or ancillary.

### Self-reported experiences of presenteeism

Of the 1,776 people who responded to the question, 1,579 (88.9%) indicated that they had attended work while feeling unwell at least once or twice in the three months before completing the survey; 93 (5.2%) reported presenteeism on most days during that period. 1,168 (68.3%) of the 1,711 respondents considered that presenteeism was relevant or very relevant to their team; 922 (53.9%) of the 1,709 respondents indicated that working when unwell is expected or fairly common and generally accepted in their team.

Of the 1,703 participants who responded, 785 (46.1%) reported feeling under pressure to work when not well. Qualitative responses indicated that this pressure frequently came from employees themselves and related to not wanting to let people (patients or colleagues) down, or not wanting to leave their colleagues with additional work.

When asked about the impact of other members of the team attending work while unwell, 733 (40.1%) of the 1,828 respondents indicated multiple impacts. The reported impacts are outlined in Table 3. The most commonly reported impacts were outlined as: feeling concern about the colleague who was unwell (*n* = 1,056; 57.8%), subsequently becoming unwell themselves and either attended work while unwell (*n* = 753; 41.2%) or have taken sickness absence (*n* = 428; 23.4%). When asked to expand on concerns about colleagues, respondents (*n* = 1,028) indicated specific concerns related to their colleague's wellbeing (*n* = 923; 89.8%), catching an illness from the colleague (*n* = 654; 63.6%), patients catching an illness from the colleague (*n* = 514; 50.0%), and tasks being completed properly (*n* = 439; 42.7%) were all frequently indicated, with 782 participants (76.1%) reporting multiple concerns related to their colleagues. Another impact noted was that participants' work had been affected by colleagues attending work when unwell (*n* = 313; 17.1%); mainly this was related to duties being changed to cover tasks their unwell colleague was not able to complete (*n* = 148; 47.3%) or needing to work alongside their colleague to share responsibility for completing tasks (*n* = 133; 42.2%). Qualitative responses highlighted that adjustments to working practices such as working remotely rather than in the shared office/ward environment did not eliminate the effects of presenteeism on other team members. Instead, responses indicated that: “*Colleagues have “worked from home” rather than come into the office when unwell, leaving more work for the person in the office.” (Respondent 1209).*

**Table 3:** Impact of team members attending work while unwell and additional details reported by the total sample and sub-samples (*N* and percentage).

Reported Impact	Number of respondents
Additional details	*N* (%), sample
My work has been affected by other people attending work whilst being unwell My duties have been changed to cover tasks my colleague(s) are not able to complete I have worked alongside my colleague(s) to share the responsibility for completing tasks	313 (17.1), 1,828 148 (47.7), 310 133 (42.9), 310
I have subsequently been unwell and as a result have come to work feeling unwell	753 (41.2), 1,828
I have subsequently been unwell and been absent from work due to being unwell	428 (23.4), 1,828
I have felt concerned about my colleagues working when they have been unwell I have felt concerned about my colleague(s) wellbeing I have felt concerned about catching an illness from my colleague(s) while at work I have felt concerned about patients catching an illness from my colleague(s) I have felt concerned about tasks getting completed properly when someone is unwell at work	1,056 (57.8), 1,828 923 (36.0), 1,028 654 (25.5), 1,028 514 (20.1), 1,028 439 (17.1), 1,028
I have not been affected by my colleagues working when they have been unwell	331 (18.1), 1,828

Full sample *N* = 1,828.

The proportion of respondents who felt confident making decisions about whether to attend work when they felt unwell was 64.6% (*n* = 1,126). When making decisions about attending work when feeling unwell, 1,608 of the 1,746 respondents (92.1%; 95% CI = 90.7%, 93.3%) participants indicated multiple considerations. The most common considerations included: the severity of the problem (*n* = 1,398; 80.1%; 95% CI = 78.1%, 81.9%), whether symptoms might be contagious (*n* = 1,265; 72.5%; 95% CI = 70.3%, 74.5%), whether they would be able to complete their expected tasks (*n* = 1,176; 67.4%; 95% CI = 65.1%, 70.0%), and the level of staffing in the team (*n* = 1,139; 65.2%; 95% CI = 62.9%, 67.5%). Qualitative responses highlighted that many participants felt a sense of guilt when making decisions about attending or being absent from work when unwell: “*I think people continue to attend work when unwell as they feel they are letting down their patients and, depending on the length of sick leave, are placing added pressure to already stressed colleagues (Respondent 1526).* There were indications that respondents would value some guidance about making these decisions: *It would be nice to know where we stand as an organisation, there seems to be a policy that we have to come in which spreads bugs when we could wfh [work from home]. Some can wfh some have to call in sick.” (Respondent 97).*

### Differences in self-reports of presenteeism when considering long-term conditions and line management responsibilities

We have no evidence of a difference in the frequency of presenteeism between people with and without line management responsibilities [Pearson Chi-square(4) = 3.209, *p* = 0.525]; however, a statistically significant difference was noted for people with and without long-term conditions [Pearson Chi-square(4) = 162.194, *p* < 0.001]. A higher proportion of people with long-term conditions reported attending work while unwell “more than 10 times” (long-term conditions: *n* = 132, 17.5%, 95% CI = 14.9%–20.4% of the 753 people reporting long-term conditions vs. no long-term conditions: *n* = 57, 6.1%, 95% CI = 4.6%–7.8% of the 943 responses from those with no long-term conditions) or “on most days” (long-term conditions: *n* = 74, 9.8%, 95% CI = 7.8%–12.2% vs. no long-term conditions: *n* = 15, 1.6%, 95% CI = 0.9%–2.6%) during the three months prior to survey completion. In contrast, those without long-term conditions more frequently reported attending work while unwell “once or twice” (long-term conditions: *n* = 255, 33.9%, 95% CI = 30.5%–37.4% vs. no long-term conditions: *n* = 462, 48.9%, 95% CI = 45.8%–52.2%) or not at all (long-term conditions: *n* = 39, 5.2%, 95% CI = 3.7%–7.0% vs. no long-term conditions: *n* = 135, 14.3%, 95% CI = 12.1%–16.7%) during the same period.

The results show no evidence of a difference in respondent opinions about whether presenteeism is relevant to their team between people with and without line management responsibilities [Pearson Chi-square(4) = 10.531, *p* = 0.032]; however significant differences were noted for people with and without long-term conditions (Very relevant/Relevant combined: long-term conditions *n* = 533, 72.2%, 95% CI = 68.8%–75.4% of the 738 respondents with long-term conditions, vs. no long-term conditions *n* = 614, 65.2%, 95% CI = 62.1%–68.3% of the 941 respondents without long-term conditions; Not relevant/Not at all relevant combined: long-term conditions *n* = 65, 8.8%, 95% CI = 6.9%–11.1% vs. no long-term conditions *n* = 127, 13.5%, 95% CI = 11.4%–15.8%; Pearson Chi-square(4) = 42.337, *p* < 0.001). In this instance, a larger proportion of people with long-term conditions felt that presenteeism was relevant, compared to those without long-term conditions.

We found no evidence of a difference in the acceptability of presenteeism, between those with and without line management responsibilities [Pearson Chi-square(4) = 5.122, *p* = 0.275]. In contrast, those with and without long-term conditions did view the acceptability of presenteeism within their team differently [Pearson Chi-square(4) = 46.206, *p* < 0.001]. A larger proportion of the 738 respondents with long-term conditions considered presenteeism to be expected and that their team relied upon people attending work while unwell than did the 939 respondents without long-term conditions (long-term conditions: *n* = 80, 10.8%, 95% CI = 8.7%–13.3% vs. no long-term conditions: *n* = 36, 3.8%, 95% CI = 2.7%–5.3%), or that presenteeism was fairly common and generally accepted within their team (long-term conditions: *n* = 362, 49.1%, 95% CI = 45.4%–52.7% vs. no long-term conditions: *n* = 431, 45.9%, 95% CI = 42.7%–49.14%). A larger proportion of people without long-term conditions viewed presenteeism as not the norm within their team (Not the norm, Not common or accepted and Highly uncommon and unacceptable combined: long-term conditions: *n* = 207, 28.0%, 95% CI = 24.8%–31.4% vs. no long-term conditions: *n* = 370, 39.4%, 95% CI = 36.3%–42.6%).

### Experiences of noticing presenteeism in the workplace

The vast majority of survey respondents indicated that they notice when colleagues attend work unwell (*n* = 1,499; 90.4%); in contrast, only 54.7% (*n* = 903) indicated that their colleagues noticed when they attended work unwell. Physical signs of illness (coughing, sneezing, limping, or other visible signs; *n* = 1,421, 95.8%), changes in interactions with colleagues (*n* = 949; 63.9%) or patients (*n* = 287; 19.3%) such as being quieter/more sensitive than usual and changes in working practices (swapping duties or remote working; *n* = 357, 24.1%) were the most common signs of presenteeism. 1,000 participants (67.4%) reported noticing more than one sign of presenteeism in their colleagues. When participants were asked how they knew that their colleagues had noticed they were unwell, fewer than half of participants responded (*n* = 897; 48.5%). Of those who did respond, the most commonly identified indications were verbal, such as colleagues asking if they were feeling ok (*n* = 871; 97.1%), colleagues offering to help with tasks the respondent was struggling with (*n* = 285; 31.8%) or managers/supervisors initiating a conversation (*n* = 228; 25.4%). 382 of these participants (42.6%) reported more than one indication that their colleagues had noticed they were unwell at work.

### Support relating to presenteeism in the workplace

When asked about more formal processes for identifying presenteeism and providing support to employees, respondents largely indicated that they were unaware of processes for identifying individuals who attended work when unwell (*n* = 1,398; 85.1%). Qualitative responses suggested that people are responsible for identifying themselves; this was especially true for lone workers: “*I would have to email as I don't see them [manager]. They are usually most reliably contactable this way. I would also speak to a colleague from my team (usually phone as often work alone).*” *(Respondent 645).*

A large proportion of participants felt well supported by their line manager and could ask for advice about attending work (*n* = 1,140; 68.5%). Although nearly half of respondents were not aware of any support for presenteeism within their workplace (*n* = 765; 47.1%), most considered that their line manager/supervisor was responsible for offering support (*n* = 742; 95.5%) and the majority felt that this was appropriate (*n* = 1,939; 62.1%). In total, 562 respondents indicated that ad hoc conversations, either in the usual workplace (*n* = 219; 39.0%) or in a private space within the usual work environment (*n* = 376; 66.9%), or planned meetings (*n* = 268; 47.7%) were reported to be the most common ways that employees were approached about support for presenteeism; conversations in a private space or planned meetings (*n* = 983, 67.9%; *n* = 796, 55.0% respectively) were considered by 1,447 respondents to be the most appropriate ways to approach an employee to offer support related to presenteeism.

Within the free-text responses, the NHS Trust sickness policy and procedures were highlighted by many respondents as influencing presenteeism. Qualitative responses to various survey questions highlighted that employees were concerned about taking sickness absence and consequences for disciplinary procedures or job security: “*I have had to utilise sick leave before but returned before being fully well due to fear of losing my job*.” *(Respondent 378).*

Although some employees reported the ability to make adjustments to their role (working remotely, changing hours/tasks, altering the mode of appointment or meetings from in person to online) this was not possible for all respondents: “*I was ill at work 3 times within 12 months, got a warning, but I work on maintenance so not possible to work from home. I think this is a bit unfair, I turned up to work and was really ill and had to go home*.” *(Respondent 1683).*

Additionally, individuals who reported experiencing long-term conditions indicated specific problems with the staff sickness policy which negatively affected those with pre-existing health conditions: “*Giving someone formal sickness meetings when they have a long-term health condition makes if feel extremely humiliating, leading to increased anxiety about being unwell and becoming more depressed due to feeling bad they you have had enough time off to warrant a formal sickness meeting, when its something that cannot be helped and investigations are ongoing*.” Responses suggested that a more flexible approach: “*a personalised sickness policy would be helpful as staff could take the necessary time to attend to their wellbeing and relax in the knowledge that they won't face recrimination.” (Respondent 1666).*

Awareness of individual employee needs relating to their long-term conditions were also highlighted as challenging: “*I think long term health conditions are often overlooked especially in periods of instability with either treatment changes or relapses. Changes in staff or team coordinators means a loss of information of previous occupational health recommendations and ways of working. It's like starting from scratch again. It's hard to keep going back over what has happened for new colleagues. I think this is an area that needs work. If someone with a long-term health issues can achieve decent balance by reducing hours, it doesn't mean that the health issue is resolved. There are still days where I don't feel well at work.” (Respondent 1133).*

### Additional analyses—generational differences

During the qualitative analysis of survey data, some responses indicated the potential for an additional factor which might affect the way experiences of presenteeism are viewed by NHS employees: cultural differences which were based on generational expectations of work. This challenge was framed as something which may require specific responses for employees of different generational expectations: “*We currently have staff with 30+*  *years experience who qualified into a system where, unless you couldn't get out of bed, you attended work and got on with it, working alongside a new generation of staff who understand their rights to a safe and healthy work environment. Plus all the generations in between! There is a lot of work still to do in normalising getting sick/unwell and how short-term care or some reasonable adjustments can prevent long-term sickness and burnout. But there is also work needed to promote resilience and a sense of community responsibility that acknowledges low level illness but encourages action to avoid disruption to work and lots of short periods of sickness.” (Respondent 1363).*

As a result, we conducted additional analyses to ascertain differences in frequency and views about presenteeism for respondents in different age categories. We found no evidence of a difference in the reported frequency of self-reported presenteeism between people in different age categories (Pearson Chi-square(12) = 22.450, *p* = 0.033). Additionally, we have no evidence of differences in respondent views about the relevance of presenteeism to their team, dependent on their age (Pearson Chi-square(12) = 15.390, *p* = 0.221). Finally, we explored the acceptability of presenteeism within teams, dependent on age. Again, our results show no evidence of differences between responses were noted (Pearson Chi-square(12) = 9.168, *p* = 0.689).

### Future research considerations

At the end of the survey, 44.4% (*n* = 811) provided their email address to be entered into a prize draw and 31.6% (*n* = 577) to be contacted about further research about presenteeism and staff wellbeing.

## Discussion

This survey of NHS employees working within a Trust based in the North of England indicated that self-reported experiences of presenteeism are common and perceived as relevant to the majority of respondents. No evidence of differences was noted between those with and without line management responsibilities, or individuals from different age groups. However, differences were noted between those with and without long-term conditions in relation to the frequency, relevance and acceptability of presenteeism.

Staff sickness policies and related disciplinary procedures were highlighted by respondents as creating a sense of fear among employees who recognise they are unwell but worry about consequences of taking time off work to fully recover from illness. This finding aligns with previous research which has also highlighted that fears of employment consequences, along with other personal factors such as decreased self-rated health, are associated with presenteeism (24). Suggestions from respondents were to create a more personalised approach rather than applying a blanket sickness policy to all employees. This personalised approach would take account of any long-term condition and may enable employers to demonstrate adherence to the Equality Act 2010 which outlines the requirement for reasonable adjustments to be made to ensure people are not disadvantaged in the workplace due to disabilities or health conditions. In addition, where an employee's role is unable to be adjusted to account for illness or injury, additional flexibility could be incorporated into this more personalised approach to sickness absence. While this would not be required under the Equality Act, recognising the potential disadvantage to employees working in roles which cannot be adapted to remote work or more flexible hours and mitigate this issue within a personalised sickness policy may support improved employee wellbeing. Further research to develop and evaluate the impact of a personalised sickness policy would be beneficial.

The level of staff engagement with this survey was high, with nearly 20% of employees contributing to this research (25, 26). This response rate was attributed to individualised emails which were sent to each employee containing the anonymous survey link which was used by over 90% of respondents. With over 30% of respondents indicating willingness to be contacted about further research on this topic, this survey indicates that primary research into employee wellbeing and presenteeism is feasible with NHS environments. The option for respondents to be entered into a prize draw to thank them for their time and contributions was considered to be successful, with over 40% respondents entering the draw; however, this was not considered to incentivise participation as the majority of respondents participated without entering the draw.

This survey captured data from a wide range of employees, with diversity in gender, age, ethnicity, setting of employment and role-type. This variety in respondents indicates that the findings can be considered relevant to, although not necessarily representative of, the workforce within this NHS Trust. Repetition of this survey in other NHS environments would provide more generalisable findings. Additionally, this survey allowed employees to express their opinions, anonymously and independently of NHS data collectors. Our findings show higher levels of both self-reported presenteeism and long-term conditions than were reported for the same NHS Trust during the NHS Staff Survey. This may reflect the difference in response rates between these surveys and self-selection bias within the current sample. Differences in survey responses would be worth exploring in future research, to better understand variables which affect the reliability and interpretation of results provided by employees.

It is worth noting that this survey contained study-specific questions which were designed to explore the relevance and acceptability of employee wellbeing and presenteeism in NHS settings. While these were developed in collaboration with our NHS partners to ensure relevance to the organisation, there are a number of validated questionnaires which capture employee wellbeing and presenteeism information and use of those validated tools would have provided findings which could be compared across workforces and environments (27). These more formal questionnaires were not included in this survey to maximise brevity while providing an indication of the importance and interest in research on this topic within this NHS Trust setting. Although the lack of ability to compare with other research could be considered a weakness of the present study, the evidence of employee support for presenteeism research generated from this survey indicates that further research incorporating validated measures of employee wellbeing and presenteeism would be feasible and would complement the current findings. Additionally, a power analysis for sample size calculation was not used to identify a target sample size, which could be considered a limitation. A strength of the survey design included the ability for respondents to complete questions over multiple sessions if required (28). In practice, this meant that a cookie was placed on the respondent's browser to track their survey progress and allowed them to continue from where they had left the survey within the recruitment period. This increased the likelihood of respondents completing the survey but did not remove the possibility of duplicate responses being made by the same respondent if a different browser and IP address were used; this is a weakness of the survey design. An additional point to consider when interpreting the results of this survey is that following consent, item completion was not mandatory. This means that survey questions were completed by subsamples of those consenting to participate. It was not within the scope of this study to understand why people may have chosen not to answer specific questions. Further work would be required to analyse the nature and reasons for non-response to specific questions; this was outside the scope of the current project, but could helpfully contribute to future efforts to explore the experiences of and opinions relating to presenteeism in the NHS workforce. Survey respondents volunteered to participate in this research and therefore the findings will be limited by response bias (29).

Employee health is changing, with more people working while living with long-term health conditions (15). Additionally, NHS environments create work settings which are challenging (4). This survey has highlighted that employee wellbeing and presenteeism research is relevant, acceptable and of interest to NHS employees. Our results indicate that further research which specifically focuses on supporting those with long-term conditions, is warranted. The findings relating to feelings of guilt when making decisions about attending work indicate that support in this area would be beneficial; this was reinforced by respondent comments explicitly requesting guidance on this topic to enable consistency in decision-making and responses to presenteeism across the workforce.

## Data Availability

The datasets presented in this study can be found in online repositories. The names of the repository/repositories and accession number(s) can be found below: anonymised data from this survey are publicly available through the Open Science Framework: https://osf.io/2hz4e/overview?view_only=ebdfeb0cccb24533b287956a75ba9c6c.
