# Supplementary material for: Prevalence and experiences of presenteeism in the workplace: a cross-sectional survey of an English National Health Service Mental Health Trust
Source: Front Health Serv. 2026 Jul 8;6:1823661. doi: 10.3389/frhs.2026.1823661 (PMC13388897; doi:10.3389/frhs.2026.1823661)
Supplement: Supplementary file 1 [file Table1.docx]

**Supplementary Information**

S1: Strobe checklist for cross-sectional studies

STROBE Statement—Checklist of items that should be included in reports of ***cross-sectional studies***

|  | Item No | Recommendation | Page |
| --- | --- | --- | --- |
| **Title and abstract** | 1 | (*a*) Indicate the study’s design with a commonly used term in the title or the abstract | 1 |
|  |  | (*b*) Provide in the abstract an informative and balanced summary of what was done and what was found | 2 |
| Introduction | | |  |
| Background/rationale | 2 | Explain the scientific background and rationale for the investigation being reported | 3 |
| Objectives | 3 | State specific objectives, including any prespecified hypotheses | 3-4 |
| Methods | | |  |
| Study design | 4 | Present key elements of study design early in the paper | 4 |
| Setting | 5 | Describe the setting, locations, and relevant dates, including periods of recruitment, exposure, follow-up, and data collection | 4 |
| Participants | 6 | (*a*) Give the eligibility criteria, and the sources and methods of selection of participants | 4 |
| Variables | 7 | Clearly define all outcomes, exposures, predictors, potential confounders, and effect modifiers. Give diagnostic criteria, if applicable | 4, S2 |
| Data sources/ measurement | 8* | For each variable of interest, give sources of data and details of methods of assessment (measurement). Describe comparability of assessment methods if there is more than one group | 4, S2 |
| Bias | 9 | Describe any efforts to address potential sources of bias | 4 |
| Study size | 10 | Explain how the study size was arrived at | 4 |
| Quantitative variables | 11 | Explain how quantitative variables were handled in the analyses. If applicable, describe which groupings were chosen and why | 5 |
| Statistical methods | 12 | (*a*) Describe all statistical methods, including those used to control for confounding | 5 |
|  |  | (*b*) Describe any methods used to examine subgroups and interactions | 5 |
|  |  | (*c*) Explain how missing data were addressed | 5 |
|  |  | (*d*) If applicable, describe analytical methods taking account of sampling strategy | N/A |
|  |  | (*e*) Describe any sensitivity analyses | N/A |
| Results | | |  |
| Participants | 13* | (a) Report numbers of individuals at each stage of study—eg numbers potentially eligible, examined for eligibility, confirmed eligible, included in the study, completing follow-up, and analysed | 5 |
|  |  | (b) Give reasons for non-participation at each stage | N/A |
|  |  | (c) Consider use of a flow diagram | N/A |
| Descriptive data | 14* | (a) Give characteristics of study participants (eg demographic, clinical, social) and information on exposures and potential confounders | Tables 1 & 2 |
|  |  | (b) Indicate number of participants with missing data for each variable of interest | Tables 1 & 2 |
| Outcome data | 15* | Report numbers of outcome events or summary measures | N/A |
| Main results | 16 | (*a*) Give unadjusted estimates and, if applicable, confounder-adjusted estimates and their precision (eg, 95% confidence interval). Make clear which confounders were adjusted for and why they were included | 7-11, Table 3 |
|  |  | (*b*) Report category boundaries when continuous variables were categorized | N/A |
|  |  | (*c*) If relevant, consider translating estimates of relative risk into absolute risk for a meaningful time period | N/A |
| Other analyses | 17 | Report other analyses done—eg analyses of subgroups and interactions, and sensitivity analyses | 9 |
| Discussion | | |  |
| Key results | 18 | Summarise key results with reference to study objectives | 11-12 |
| Limitations | 19 | Discuss limitations of the study, taking into account sources of potential bias or imprecision. Discuss both direction and magnitude of any potential bias | 12 |
| Interpretation | 20 | Give a cautious overall interpretation of results considering objectives, limitations, multiplicity of analyses, results from similar studies, and other relevant evidence | 13 |
| Generalisability | 21 | Discuss the generalisability (external validity) of the study results | N/A |
| Other information | | |  |
| Funding | 22 | Give the source of funding and the role of the funders for the present study and, if applicable, for the original study on which the present article is based | 13 |

*Give information separately for exposed and unexposed groups.

S2: Survey questions

Demographic information

1. What is your gender? (Female/ Male/ Non-binary/ Prefer to self-describe/ Prefer not to say)
2. What is your age in years? (16-20/ 21-30/ 31-40/ 41-50/ 51-65/ 65+)
3. What is your ethnic group? (White/ Mixed or Multiple ethnic background/ Asian or Asian British/ Black or African or Caribbean or Black British/ Other ethnic group)
4. What type of service do you work in? Please select all that apply. (Inpatient/ Outpatient/ Community/ Office-based/ General buildings and estates)
5. What is your occupational group? Please select all that apply. (Allied Health Professionals or Healthcare Scientists or Scientific and Technical/ Medical and Dental/ Ambulance (operational)/ Public Health/ Commissioning/ Registered Nurses and Midwives/ Nursing or Healthcare Assistants/ Social Care/ Wider Healthcare Team including: admin and clerical, central functions and corporate services, maintenance or ancillary)
6. Does part of your role involve the line-management of other employees? (Yes/ No)
7. How long have you worked for [NHS Trust name]? (Less than 1 year/ 1-5 years/ 6-9 years/ 10+ years)

Experiences of presenteeism

1. Thinking about the last three months at work, which of the following describes you best? (I didn’t go to work feeling unwell/ I went to work feeling unwell once or twice/ I went to work feeling unwell on several occasions (more than 3 times)/ I went to work feeling unwell many times (more than 10 times)/ I went to work feeling unwell on most days)
2. When you feel unwell and are due to attend work, what do you consider before deciding whether to attend work? Please select all that apply. (The severity of the problem (illness/symptom of a health condition)/ Whether your symptoms might be contagious/ Whether you will be able to complete the tasks expected of you/ Whether anyone else will be able to complete the tasks on your behalf/ The level of staffing in the team/ Previous experience of attending when feeling unwell/ Previous absences / sickness record/ Other, please describe)
3. Please indicate your level of agreement with the following statement: *'I feel confident in making decisions about whether to go to work or not when I am unwell.'*(Strongly agree/ Agree/ Neither agree nor disagree/ Disagree/ Strongly disagree)
4. We understand that many people with a long-term health condition might experience presenteeism differently to those who experience temporary illnesses.  A long-term health condition is defined as a condition that cannot at present be cured but can be controlled by medication and therapies.  Do you have a long-term health condition? (Yes/ No/Prefer not to say)
   1. If yes, Does this ever affect your ability to attend work? (Yes/ No/ Prefer not to say)
   2. If yes, Does this ever affect your ability to carry out your duties at work? (Yes/ No/ Prefer not to say)
   3. If yes, Do you ever attend work while feeling unwell for reasons not relating to your long-term condition? (Yes/ No/ Prefer not to say)
   4. If yes, Do you ever feel that your ability to carry out your duties is affected by illnesses which are not related to your long-term condition? (Yes/ No/ Prefer not to say)
5. Would you say that working when unwell is a topic that is relevant to your team? (Yes, very relevant/ Yes, relevant/ Not sure/ Not really relevant/ No, it’s not relevant at all)
   1. If Yes, very relevant/ Yes, relevant, Please tell us how presenteeism is relevant to your team
6. Please complete the following statement using the suggestions provided: *'Working when unwell is….’* (expected in our team - we rely on people attending work/ fairly common and generally accepted in our team/ not the norm in our team/ not common or accepted in our team/ highly uncommon and unacceptable in our team/ prefer not to say)
7. Please indicate whether you agree with the following statement: *‘I often feel under pressure to work when not well’* (Strongly agree/ Agree/ Neither agree nor disagree/ Disagree/ Strongly disagree)
8. What is the impact of other members of your team working when they are unwell? Please select all that apply. (My work has been affected by other people attending work whilst being unwell/ I have subsequently been unwell and as a result have come to work feeling unwell/ I have subsequently been unwell and been absent from work due to being unwell/ I have felt concerned about my colleagues working when they have been unwell/ I have not been affected by my colleagues working when they have been unwell/ Prefer not to say, Other, please describe)
   1. If My work has been affected by other people attending work whilst being unwell, Please tell us how your work has been affected: (My duties have been changed to cover tasks my colleague(s) are not able to complete/ I have worked alongside my colleague(s) to share the responsibility for completing tasks/ Other, please describe)
   2. If I have felt concerned about my colleagues working when they have been unwell, Please tell us about your concerns:  Please select all that apply. (I have felt concerned about my colleague(s) wellbeing/ I have felt concerned about catching an illness from my colleague(s) while at work/ I have felt concerned about patients catching an illness from my colleague(s)/ I have felt concerned about tasks getting completed properly when someone is unwell at work/ Other, please describe)
9. Please indicate your level of agreement with the following statement: *‘I feel well supported by my line manager and know that I can seek advice from them about whether to attend work or not’*  (Strongly agree/ Agree/ Neither agree nor disagree/ Disagree/ Strongly disagree)

Noticing presenteeism in the workplace

1. Do you notice when your colleague(s) attend work when they are unwell? (Yes/ No/ Prefer not to say)
   1. If yes, What are the first signs of presenteeism that you notice?  Please select all that apply. (Physical signs of illness such as coughing or sneezing, limping, visual discomfort/ Change in the way my colleague(s) interact with other staff members/ Change in the way my colleague(s) interact with patients/ Change in work such as request to swap duties/ Other, please describe)
2. Do other people notice if you attend work when you are unwell? (Yes/ No/ Prefer not to say)
   1. If yes, How do you know that your colleague(s) have noticed you working when you are unwell?  Please select all that apply. (Someone asks me if I’m feeling ok or unwell/ Someone offers to help with a task I’m struggling with/ My manager/supervisor speaks with me/ Other, please describe)
3. Are you aware of a process in your workplace which helps to spot people who are attending work while they are unwell? (Yes/ No/ Prefer not to say)
   1. If Yes/ Prefer not to say, How are people who attend work when they are unwell currently identified? (People are responsible for identifying themselves to managers/supervisors/ Managers/supervisors ask team members during their working day/ People who attend work while they are unwell are not currently identified/ Not sure/ Other, please describe)
4. If you were at work and feeling unwell, what would be the most acceptable way for your manager/supervisor to become aware of this? (I would prefer to initiate a conversation with my manager or supervisor/ I would prefer my manager or supervisor to ask me/ I don’t think it’s necessary for my manager/supervisor to know that I’m feeling unwell/ Not sure/ Other, please describe)

Support options relating to presenteeism in the workplace

1. Which of the following best describes the current situation in your workplace? (There is a lot of support available for presenteeism/ There is some support available for presenteeism/ There isn’t much support for presenteeism/ I’m not aware of any support available for presenteeism/ Not sure)

NOTE: If I’m not aware of any support available for presenteeism, SKIP TO QUESTION 26

1. Please tell us about the support available which promotes employee wellbeing and is relevant to presenteeism
2. Who is currently responsible for offering support related to presenteeism to members of your team?  Please select all that apply. (Overall team manager or supervisor/ On duty manager or supervisor/ Colleague/ Member of the HR team/ Organisation external to our Trust/ Support is not currently offered/ Other, please describe/ Not sure)
3. How are employees in your team currently approached about support relating to presenteeism?  Please select all that apply. (Ad hoc conversation in usual workspace/ Ad hoc conversation in private but in usual work environment/ Arrange a meeting specifically to discuss this in person or via telephone or via video/ Email or letter/ People in my team are not currently approached about support relating to presenteeism/ Other, please describe/ Not sure)
4. Do you feel that there is enough support in your work context to promote employee wellbeing related to presenteeism? (Yes/ No/ Not sure)
   1. If no, Please tell us about additional support related to presenteeism which you would like to be available to members of your team
5. Ideally, who do you think should be responsible for offering support related to presenteeism? Please select all that apply. (Overall team manager or supervisor/ On duty manager or supervisor/ Colleague/ Member of the HR team/ Organisation external to our Trust/ Support relating to presenteeism is not needed in my team / Other, please describe)

NOTE: If Support relating to presenteeism is not needed in my team, SKIP TO QUESTION 28

1. Ideally, what do you think is the best way for someone to approach an individual about support relating to presenteeism? Please select all that apply. (Ad hoc conversation in usual workspace/ Ad hoc conversation in private but in usual work environment/ Arrange a meeting specifically to discuss this in person or via telephone or via video/ Email or letter/ Support related to presenteeism is not needed in my team / Other, please describe)
2. Please use the following box to tell us if you would like any more information about this topic or if you have any concerns about future research on this topic.
